# Clinicopathological features of early stage gastric adenocarcinoma of fundic gland type: Case series

**DOI:** 10.1097/MD.0000000000028469

**Published:** 2022-01-14

**Authors:** Huan Zhang, Shuyan Wang, Yongping Zhang, Fusang Ye, Chunnian Wang

**Affiliations:** Department of Pathology, Ningbo Diagnostic Pathology Center, Ningbo, Zhejiang, China.

**Keywords:** gastric adenocarcinoma of fundic gland type, H+/K+-ATPase, pepsinogen-I

## Abstract

**Introduction:**

Gastric adenocarcinoma of the fundic gland type (GA-FG) is characterized by a well-differentiated neoplasm. More than 100 cases have been reported, but only a few cases have been described in China. Therefore, its clinicopathological characteristics need to be investigated further. Herein, we report five cases and briefly review the relevant literature.

**Patient concerns:**

Five patients, including three women and two men, were identified in the Ningbo Clinical Pathological Diagnosis Center between March 2017 and July 2020. Patients (case 1, case 2, and case 5) underwent gastroscopy due to epigastric pain. Apart from the lesion, others were occasionally discovered on physical examination.

**Diagnosis:**

Gastric adenocarcinoma of the fundic gland type (GA-FG).

**Intervention:**

Five patients were treated with endoscopic submucosal dissection.

**Outcomes:**

Surgical outcomes were good. Esophagogastroduodenoscopy showed a scar with no recurrence, and no postoperative symptoms were observed from 3 to 43 months during the follow-up.

**Conclusion:**

We present five cases of well-differentiated tubular adenocarcinoma that mimicked the fundic glands. Cell differentiation by MUC2, MUC5AC, MUC6, pepsinogen-I, and H+/K+-ATPase. Immunohistochemical findings in GA-FG suggested differentiation of the fundic glands. In addition, it has a low proliferation. p53 and Her-2 were negative, and β-catenin was positive in the cytoplasm, indicating that the pathogenesis of this tumor was different from that of traditional intestinal and diffuse gastric carcinomas. In summary, this neoplasm is rare and unusual. To better understand this issue, similar cases should be monitored in the future.

## 1 Introduction

Gastric adenocarcinoma of the fundic gland type (GA-FG) is a novel entity. In 2007, a case of GA-FG was first reported by Tsukamoto.^[1]^ Subsequently, Ueyama proposed 10 cases and proposed gastric adenocarcinoma of the fundic gland (chief cell predominant type: CCP) as a new entity of gastric adenocarcinoma in 2010.^[2]^ Since then, an increasing number of cases have been reported in Japan^[3–12]^ and Korea.^[13]^ GA-FG is distinct from traditional intestinal and diffuse gastric carcinomas. It is a well-differentiated neoplasm with unclear etiopathogenesis and has a good prognosis, rarely demonstrating metastasis and recurrence. The malignant potential of this lesion remains considerable.^[11]^ Most cases undergo endoscopic submucosal dissection (ESD), but there are few reports of surgical resection existence.^[14,15]^ GA-FG is now listed separately as a new category of gastric adenocarcinoma in the fifth edition of the World Health Organization (WHO) classification of digestive system tumors. In previous reports, GA-FGs were found to be small tumors <1 cm in diameter, and developed in the upper and middle third of the stomach.^[2]^ Macroscopically, GA-FG is a whitish tumor accompanied by irregular vascular growth, and most cases are detected as small submucosal tumor (SMT)-like lesions or whitish depressed lesions that develop in non-atrophic oxyntic mucosa.^[16]^

As is known to all, human stomach carcinomas were classified into two major groups, namely intestinal and diffuse types of Lauren in histologically. It is widely believed that the phenotypic expression of tumor cells reflects the tissue of origin.^[17]^ In previous studies, GA-FG typically displayed the expression of pepsinogen-I and H+/K+-ATPase.^[2,13,18]^ Pepsinogen-I is produced only by the chief and mucus neck cells in the fundic glands. Normal gastric parietal cells possess an H+/K+-ATPase proton pump. This enzyme is mainly located near the cell surface membranes and in the membranes of intracytoplasmic canaliculi. Therefore, H+/K+-ATPase is considered a marker of parietal cell differentiation.^[19]^ In this study, cell differentiation, mucin proteins, pepsinogen-I, and H+/K+-ATPase were evaluated.

Furthermore, we determined that Her-2 protein expression was used to assess the malignant biological behavior and prognosis of gastric cancer. Gastric cancer patients who exhibit Her-2 protein overexpression might be potential candidates for new adjuvant therapies, involving the application of humanized monoclonal antibodies.^[20]^ However, the expression of Her-2 in GA-FG is unknown. A few reports presented β-catenin and was increased in GA-FG, suggesting that Wnt/β-catenin signaling activation may be associated with tumorigenesis.^[21]^ We then collected five cases that illustrated well-differentiated tubular adenocarcinoma and mimicked the fundic glands, along with a brief review of the literature.

## 2 Case description

Characterized by well-differentiated columnar cells mimicking fundic gland cells, and notably chief cells, five cases were identified in the Ningbo Clinical Pathological Diagnosis Center between March 2017 and July 2020.

**Figure 1:**
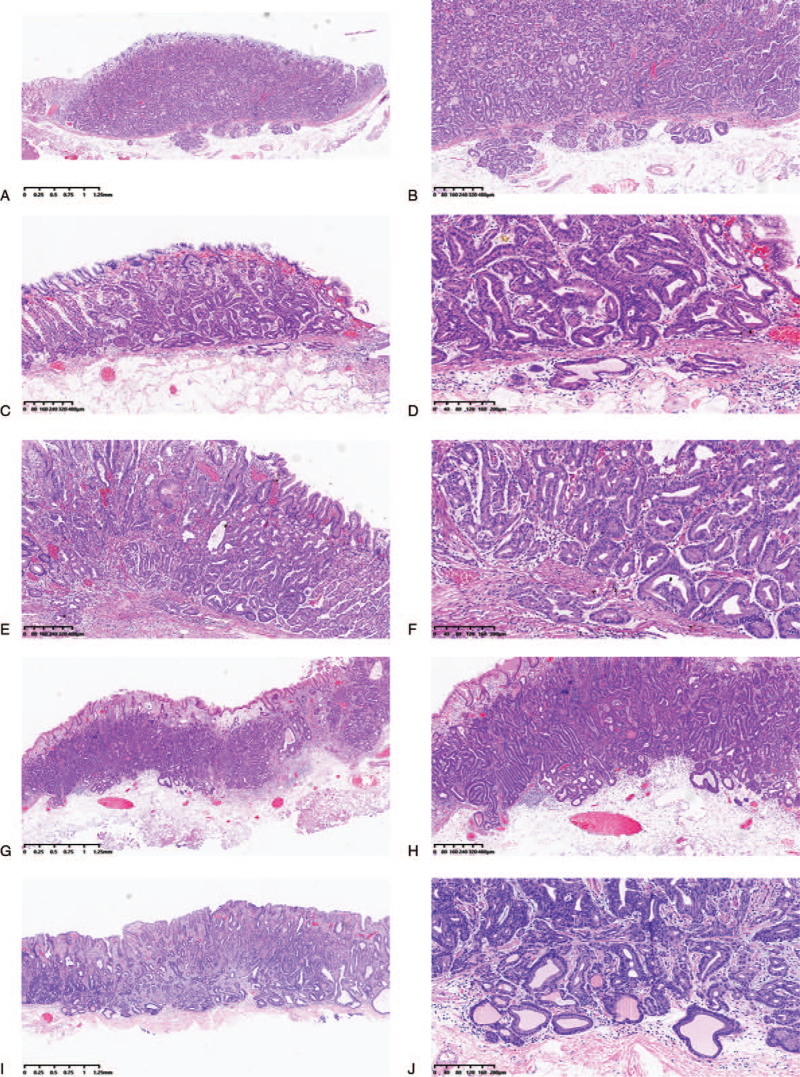
Hematoxylin and eosin stain: case 1 (A and B), case 2 (C and D), case 3 (E and F), case 4 (G and H), and case 5 (I and J). All cases display the invasion of the submucosal layer and reveal carcinoma mimicking fundic glands with irregular glandular structure.

The clinicopathologic findings are summarized in Table 1. Patients included three females and two males, aged 54–62 years (average, 57 years). Patients (case 1, case 2, and case 5) underwent gastroscopy due to epigastric pain. Apart from the lesion, others were occasionally discovered on physical examination. None of these patients had serum anti-H. pylori antibody. The endoscopic findings in all the cases were small. All patients underwent ESD. Four tumors were macroscopically identified as submucosal tumor (SMT)-like 0-IIa (superficial elevated type), and one was identified as 0-IIb (superficial flat type) and covered with normal colored or whitish vasodilated mucosa. They were small, with the diameter of 0.8, 0.5, 0.3, 0.9, and 0.8 cm (average, 0.66 cm). All cases had lesions that invaded the submucosal layer (Fig. 1). Lymphatic or venous invasion was not identified in any of the cases. None of the patients died or exhibited signs of disease recurrence during the follow-up period.

**Table 1:** The clinicopathological findings of this study.

Parameters	Case 1	Case 2	Case 3	Case 4	Case 5
Age (years)/sex	62/female	54/female	57/female	58/male	54/male
Serum anti-*H. pylori* antibody	Negative	Negative	Negative	Negative	Negative
Chronic gastritis	(–)	(–)	(–)	(–)	(–)
Therapy	ESD	ESD	ESD	ESD	ESD
Location	Upper third	Middle third	Middle third	Upper third	Lower third
Size (cm)	0.8	0.5	0.3	0.9	0.8
Gross type	0-IIa	0-IIb	0-IIa	0-IIa	0-IIa
Depth	SM (400 μm)	SM (250 μm)	SM (100 μm)	SM (200 μm)	SM (400 μm)
Lymphatic invasion	(–)	(–)	(–)	(–)	(–)
Venous invasion	(–)	(–)	(–)	(–)	(–)
Survival time (months)	43	19	16	12	3
Outcome	Alive	Alive	Alive	Alive	Alive

ESD = endoscopic submucosal dissection, SM = submucosal layer.

In addition to conventional hematoxylin and eosin staining, ESD-resected specimens were subjected to immunohistochemical staining. Histological examination of the biopsy specimens obtained from the lesion revealed that the GA-FGs in all cases were mainly composed of highly differentiated columnar cells mimicking fundic gland cells, predominantly chief cells, with pale gray-blue, basophilic cytoplasm, and mildly enlarged nuclei. In some cases, the tumor cells with coarse granular eosinophilic cytoplasm were admixed and were similar to parietal cells. With careful observation, the nuclei were slightly larger than those of normal fundic glands and were markedly hyperchromatic. The atypical glands were well-circumscribed with an abrupt transition from the normal mucosa, which was one of the signs of neoplasia. The superficial area tended to retain the normal foveolar epithelium, whereas the deep area tended to show irregular branching and dilatation. All cases of typical GA-FG are demonstrated in Figure 1.

**Figure 2:**
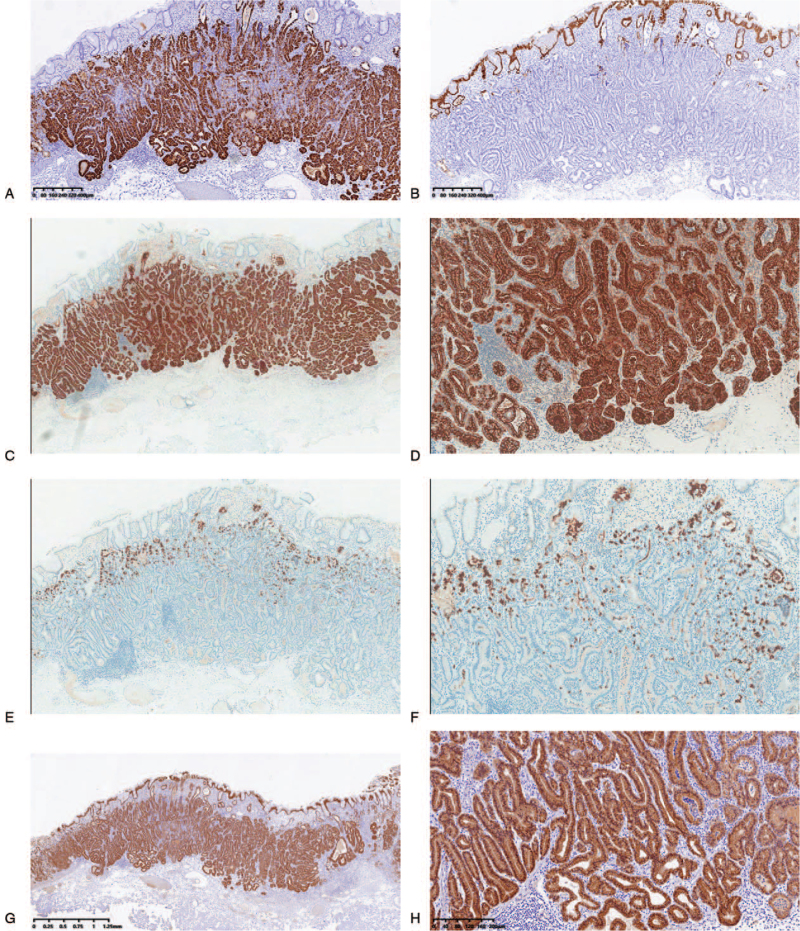
Immunohistochemical staining (EnVision) results in gastric adenocarcinoma with chief cell differentiation (case 4). Carcinoma revealed diffuse positivity for MUC6 (A), but MUC5AC was only stained in the non-atypical foveolar epithelium that was covered on the surface of tumor (B). Pepsinogen-I was strongly expressed in GAFG (C and D), and focal positivity for H+/K+-ATPase (E and F). The β-catenin of all cases was only expressed in cytoplasm (G and H).

The immunohistochemical markers MUC5AC for foveolar cells, MUC6 for mucous neck cells or pyloric gland cells, and MUC2 for goblet cells were tested. Furthermore, pepsinogen-I, a marker of differentiation to chief cells, and H+/K+-ATPase as a marker for parietal cell differentiation were also employed. When these markers were expressed in 10% or more of the cytoplasm, they were considered positive. Immunohistochemical examination revealed that the neoplastic glands were diffusely and strongly reactive for MUC6 and pepsinogen I, and nonreactive for MUC2. MUC5AC was stained only in the non-atypical foveolar epithelium that covered the tumor surface. In addition, all cases revealed focal positivity for H+/K+-ATPase. Moreover, it had a low labeling index Ki-67 (<5%). p53 and Her-2 were negative. Moreover, β-catenin was detected in the cytoplasm. Immunohistochemical discoveries of typical GA-FG are shown in Figure 2 (case 4).

## 3 Discussion

GA-FG is a recently recognized and rare pathologic subtype of gastric adenocarcinoma. However, it has distinct clinicopathological characteristics, especially in terms of tumor location, histologic features, phenotypic expression, and low-grade malignancy.^[2]^ Most reports on GA-FG cases are from Japan and Korea, with only a few reports from Western countries,^[22]^ and a few cases have been reported in China.^[23]^ In this study, we collected five cases that were identified as GA-FG. Three women and two men aged 54–62 years, indicating that this lesion often occurs in elderly people. Most previously reported cases were aged people, ranging from 42 to 82 years, while the incidence rate of males was close to that of female.^[18,24]^ In addition, the majority of cases were solitary and small, generally located in the upper third of the stomach.^[12,24,25]^ However, recently, a case of multiple gastric adenocarcinoma of fundic gland type was reported,^[26]^ and its endoscopic findings were characteristic. Endoscopy usually proves that it is covered by normal-colored or faded-whitish mucosa with vasodilatation or branched vessels on the tumor surface.^[2,3,6,16,22,23,25,27]^ Although, in some cases, endoscopy revealed chronic atrophic gastritis or intestinal metaplasia, in most cases, the tumors are surrounded by gastric mucosa without pathological evidence of mucosal changes.^[25]^ As is known to all, H pylori infection plays a major role in conventional adenocarcinoma; some previous reports demonstrated that most cases were negative in GA-FG, and all patients were negative in our report as well,^[28]^ suggesting that the pathogenesis of GA-FG may be different from conventional adenocarcinoma.

Histologically, GA-FG is a well-differentiated adenocarcinoma, mainly composed of cells resembling chief cells and is classified into CCP, parietal cell predominant type, and mixed type,^[1]^ with the most common type being CCP. However, the well-differentiated morphology of GA-FG often confuses pathologists. Previous studies have concluded the histopathological characteristics. Although GA-FG-CCP is composed of a variety of mildly atypical columnar cells that mimic the fundic glands, the atypical glands were well circumscribed with an abrupt transition from the normal mucosa.^[3,13,25]^ In addition, it varies in size and shape, with anastomosing and endless glands.^[2,3,13]^ The cytoplasm of tumor cells was pale gray to blue, basophilic, and resembled chief cells. At higher magnification, nuclei were monotonous and slightly larger than those of normal fundic gland cells, and frequently contained small but prominent nucleoli.^[13,29]^ Furthermore, the surface of the tumor is primarily covered by the non-atypical foveolar epithelium.^[2,5,25]^ The histopathology results in our cases were typical. All cases had lesions that invaded the submucosal layer (Fig. 1). Lymphatic or venous invasion was not identified in any of the cases.

At present, many studies have demonstrated that GA-FGs are characterized as neoplastic lesions that arise directly from the gastric mucosa without intervening in intestinal metaplasia, but chief cell differentiation.^[2,29]^ Hence, we confirmed that using immunohistochemical analysis. As we all know that GA-FG typically exhibited the expression of pepsinogen-I and H+/K+-ATPase, pepsinogen-I is produced by the chief and mucus neck cells in the fundic glands.^[29]^ H+/K+-ATPase is mainly located near cell surface membranes and in the membranes of intracytoplasmic canaliculi, and it is considered a marker for parietal cell differentiation. In the present study, pepsinogen-I expression was observed in all cases, thus supporting the differentiation of chief cells, which are a component of the fundic gland. All cases revealed focal positivity for H+/K+-ATPase, which was in accordance with hematoxylin and eosin staining, which showed tumor cells and resembled parietal cells. All cases were classified as GA-FG with the chief cell differentiation type in our study. In contrast, MUC2 for goblet cells was negative, and MUC5AC for foveolar cells was stained in the non-atypical foveolar epithelium that was covered on the tumor surface. In contrast, Ueyama et al speculated that MUC5AC may be expressed in advanced GA-FG-CCP lesions with a large diameter and massive submucosal invasion.^[2]^ Therefore, more cases need to be confirmed. In addition, in our report, MUC6 was also strongly expressed in GA-FG-CCP, suggesting that it is categorized as a purely gastric phenotype.

In previous reports, most reported cases of GA-FG were proven to have submucosal invasion,^[22]^ while only several cases reported lymphovascular invasion,^[2,6,12,14,15,24]^ indicating that its biological malignancy was not high. Most patients were treated with ESD.^[8,18,25,29]^ We evaluated the tumor cell proliferation ability using the Ki-67 index, which was low. As a marker of poor prognosis in gastric carcinoma, in terms of p53, the expression of all cases were negative, which was consistent with previous researches,^[2]^ which reflected that the growth of GA-FG was slow and less aggressive. It is known that the histologic features and phenotypic expression of GA-FG are different from those of conventional gastric adenocarcinoma, their pathogenesis may be distinct. In our report, we detected that Her-2 protein expression in all cases was negative and was used to assess the malignant biological behavior and prognosis of conventional gastric adenocarcinoma.^[20]^ Furthermore, β-catenin in all cases was only expressed in the cytoplasm, and the results did not correspond with those of previous studies, demonstrating that β-catenin accumulated in the nucleus, followed by activation of the Wnt/β-catenin signaling pathway. Therefore, it may be associated with tumorigenesis in GAFG.^[21]^ Thus, whether β-catenin signaling contributes to tumorigenesis in GAFG requires further investigation. In addition, several studies have indicated that GNAS mutations contribute to tumorigenesis in GAFG, and it is thought to be a common and highly specific genetic feature of GAFG. To date, the molecular mechanism of GAFG has not been clearly demonstrated.

In summary, we examined five cases of GAFG, which were rarely reported in China, and discussed their characteristics in comparison with previous reports. GAFG exhibited distinct clinicopathological characteristics compared to conventional gastric adenocarcinoma. In particular, the histologic features were deceptive, and it took a long time to understand this disease. In contrast, the tumorigenesis of GAFG has not been elucidated in detail; therefore, further studies are needed.

## Acknowledgments

Dr. Jinghong Xu is very much appreciated for the detection of pepsinogen-I and H+/+-ATPase by immunohistochemistry.

## Author contributions

**Data curation:** Huan Zhang, Chunnian Wang.

**Formal analysis:** Huan Zhang.

**Funding acquisition:** Chunnian Wang.

**Investigation:** Shuyan Wang, Yongping Zhang.

**Methodology:** Yongping Zhang, Fusang Ye.

**Writing – original draft:** Huan Zhang.

**Writing – review & editing:** Shuyan Wang, Chunnian Wang.

## References

[1] Tsukamoto T Yokoi T Maruta S. Gastric adenocarcinoma with chief cell differentiation. Pathol Int 2007;57:517–22. 17610477 10.1111/j.1440-1827.2007.02134.x

[2] Ueyama H Yao T Nakashima Y. Gastric adenocarcinoma of fundic gland type (chief cell predominant type): proposal for a new entity of gastric adenocarcinoma. Am J Surg Pathol 2010;34:609–19. 20410811 10.1097/PAS.0b013e3181d94d53

[3] Fujisawa T Ueyama S Ouchi S. Early gastric adenocarcinoma of the fundic gland type (chief cell predominant type) observed with magnifying endoscopy using narrow band imaging: report of a case. Gastroenterol Endosc 2011;53:3769–75.

[4] Miyaoka Y Izumi D Mikami H. A case report of an extremely well differentiated gastric adenocarcinoma of the fundic gland type successfully treated with ESD. Gastroenterol Endosc 2011;53:1778–85.

[5] Fujimoto Y Araki A Kaneko S. A case of gastric adenocarcinoma of fundic gland type treated by ESD. Prog Digest Endosc 2013;82:144–5.

[6] Ueyama H Matsumoto K Nagahara A Hayashi T Yao T Watanabe S. Gastric adenocarcinoma of the fundic gland type (chief cell predominant type). Endoscopy 2014;46:153–7. 24338239 10.1055/s-0033-1359042

[7] Yahata S Ouchi S Shiozawa H. Gastric adenocarcinoma of the fundic gland type occurring in mucosa that was not infected with *Helicobacter pylori*: report of a case. Gastroenterol Endosc 2014;56:1763–9.

[8] Fujii M Ishihara R Aoi K. Endoscopic features of early stage gastric adeno-carcinoma of fundic gland type (chief cell predominant type): a case report. Case Rep Clin Pathol 2015;2:17–22.

[9] Hori K Ide YH Hirota S Toyoshima F Takagawa T Nakamura S. Early gastric adenocarcinoma of the fundic gland type. Endoscopy 2015;47:E177–8. 25928827 10.1055/s-0034-1391500

[10] Isono Y Baba Y Tanaka H. Long-term follow-up of a gastric adenocarcinoma of the fundic gland type: a case report. Gastroenterol Endosc 2015;57:2639–46.

[11] Lewin E Daroca P Sikka S Wu T Nakanishi Y. Very well-differentiated gastric adenocarcinoma of the fundic gland type with intriguing morphologic features: a case report and review of the literature. Am J Clin Pathol 2015;144: A391-A. doi: 10.1093/ajcp/144.suppl2.391.

[12] Miyazawa M Matsuda M Yano M. Gastric adenocarcinoma of fundic gland type: five cases treated with endoscopic resection. World J Gastroenterol 2015;21:8208–14. 26185396 10.3748/wjg.v21.i26.8208 PMC4499367

[13] Park ES Kim YE Park CK Yao T Kushima R Kim KM. Gastric adenocarcinoma of fundic gland type: report of three cases. Korean J Pathol 2012;46:287–91. 23110017 10.4132/KoreanJPathol.2012.46.3.287 PMC3479759

[14] Okumura Y Takamatsu M Ohashi M. Gastric adenocarcinoma of fundic gland type with aggressive transformation and lymph node metastasis: a case report. J Gastric Cancer 2018;18:409–16. 30607304 10.5230/jgc.2018.18.e22 PMC6310764

[15] Yoshitake K Kumashiro Y Watanabe T. [Laparoscopic gastrectomy for gastric adenocarcinoma of the fundic gland type – report of a case]. Gan To Kagaku Ryoho 2016;43:1875–7. 28133161

[16] Chiba T Kato K Masuda T. Clinicopathological features of gastric adenocarcinoma of the fundic gland (chief cell predominant type) by retrospective and prospective analyses of endoscopic findings. Dig Endosc 2016;28:722–30. 27129734 10.1111/den.12676

[17] Tatematsu M Tsukamoto T Inada K. Stem cells and gastric cancer: role of gastric and intestinal mixed intestinal metaplasia. Dig Endosc 2003;94:135–41. 10.1111/j.1349-7006.2003.tb01409.x PMC11160206 12708487

[18] Kato M Uraoka T Isobe Y. A case of gastric adenocarcinoma of fundic gland type resected by combination of laparoscopic and endoscopic approaches to neoplasia with non-exposure technique (CLEAN-NET). Clin J Gastroenterol 2015;8:393–9. 26615600 10.1007/s12328-015-0619-2

[19] Takubo K Honma N Sawabe M. Oncocytic adenocarcinoma of the stomach: parietal cell carcinoma. Am J Surg Pathol 2002;26:458–65. 11914623 10.1097/00000478-200204000-00007

[20] Chen J Li DS Yu YH. Clinical significance of Her-2 protein expression in gastric cancer. World Chin J Digestol 2010;18:1375–9.

[21] Tajima Y Murakami T Saito T. Distinct involvement of the sonic hedgehog signaling pathway in gastric adenocarcinoma of fundic gland type and conventional gastric adenocarcinoma. Digestion 2017;96:81–91. 28738329 10.1159/000478999

[22] Benedict MA Lauwers GY Jain D. Gastric adenocarcinoma of the fundic gland type: update and literature review. Am J Clin Pathol 2018;149:461–73. 29648578 10.1093/ajcp/aqy019

[23] Jing Z Xin L Hejun Z. Clinical and pathological analysis of 11 patients with gastric adenocarcinoma of the fundic gland type. Chin J Min Inv Surg 2020;20:296–9.

[24] Kawasaki K, Kurahara K Fau - Oshiro Y, Oshiro Y Fau - Matsumoto T, Matsumoto T. Depressed Gastric Adenocarcinoma of the Fundic Gland Type. 2016(1349-7235.(Electronic)). 10.2169/internalmedicine.55.5706 26935381

[25] Miyazawa M Matsuda M Yano M. Gastric adenocarcinoma of the fundic gland (chief cell-predominant type): a review of endoscopic and clinicopathological features. World J Gastroenterol 2016;22:10523. 28082804 10.3748/wjg.v22.i48.10523 PMC5192263

[26] Shibukawa N Ouchi S Wakamatsu S Wakahara Y Kaneko A. Multiple gastric adenocarcinoma of fundic gland type after *H. pylori* eradication: a case report. Nippon Shokakibyo Gakkai zasshi [Jpn J Gastroenterol] 2020;117:245–51. 10.11405/nisshoshi.117.245 32161246

[27] Tohda G Osawa T Asada Y. Gastric adenocarcinoma of fundic gland type: endoscopic and clinicopathological features. World J Gastrointest Endosc 2016;8:244–51. 26962407 10.4253/wjge.v8.i4.244 PMC4766258

[28] Fan X Yang X-S Bai P. Gastric adenocarcinoma of the fundic gland type: a case report. Medicine 2020;99:e20361. 32481329 10.1097/MD.0000000000020361 PMC7249874

[29] Singhi AD Lazenby AJ Montgomery EA. Gastric adenocarcinoma with chief cell differentiation: a proposal for reclassification as oxyntic gland polyp/adenoma. Am J Surg Pathol 2012;36:1030–5. 22472957 10.1097/PAS.0b013e31825033e7

